# Exosomes from bone marrow mesenchymal stem cells enhance fracture healing through the promotion of osteogenesis and angiogenesis in a rat model of nonunion

**DOI:** 10.1186/s13287-020-1562-9

**Published:** 2020-01-28

**Authors:** Lu Zhang, Guangjun Jiao, Shanwu Ren, Xiaoqian Zhang, Ci Li, Wenliang Wu, Hongliang Wang, Haichun Liu, Hongming Zhou, Yunzhen Chen

**Affiliations:** 1 Department of Spine Surgery, Shandong University Qilu Hospital, Jinan, China; 2 Department of Radiology, Shandong University Qilu Hospital, Qingdao, Qingdao China; 3 Department of Spine Surgery, Linyi Central Hospital, Linyi, China

**Keywords:** Exosomes, Nonunion, Osteogenesis, Angiogenesis

## Abstract

**Background:**

As important players in cell-to-cell communication, exosomes (exo) are believed to play a similar role in promoting fracture healing. This study investigated whether exosomes derived from bone marrow mesenchymal stem cells (BMMSC-Exos) could improve fracture healing of nonunion.

**Methods:**

BMMSC-Exos were isolated and transplanted into the fracture site in a rat model of femoral nonunion (Exo group) every week. Moreover, equal volumes of phosphate-buffered saline (PBS) and exosome-depleted conditioned medium (CM-Exo) were injected into the femoral fracture sites of the rats in the control and CM-Exo groups. Bone healing processes were recorded and evaluated by radiographic methods on weeks 8, 14 and 20 after surgery. Osteogenesis and angiogenesis at the fracture sites were evaluated by radiographic and histological methods on postoperative week 20. The expression levels of osteogenesis- or angiogenesis-related genes were evaluated in vitro by western blotting and immunohistochemistry. The ability to internalize exosomes was assessed using the PKH26 assay. Altered proliferation and migration of human umbilical vein endothelial cells (HUVECs) and mouse embryo osteoblast precursor cells (MC3TE-E1s) treated with BMMSC-Exos were determined by utilizing EdU incorporation, immunofluorescence staining, and scratch wound assay. The angiogenesis ability of HUVECs was evaluated through tube formation assays. Finally, to explore the effect of exosomes in osteogenesis via the BMP-2/Smad1/RUNX2 signalling pathway, the BMP-2 inhibitors noggin and LDN193189 were utilized, and their subsequent effects were observed.

**Results:**

BMMSC-Exos were observed to be spherical with a diameter of approximately 122 nm. CD9, CD63 and CD81 were expressed. Transplantation of BMMSC-Exos obviously enhanced osteogenesis, angiogenesis and bone healing processes in a rat model of femoral nonunion. BMMSC-Exos were taken up by HUVECs and MC3T3-E1 in vitro, and their proliferation and migration were also improved. Finally, experiments with BMP2 inhibitors confirmed that the BMP-2/Smad1/RUNX2 signalling pathway played an important role in the pro-osteogenesis induced by BMMSC-Exos and enhanced fracture healing of nonunion.

**Conclusions:**

Our findings suggest that transplantation of BMMSC-Exos exerts a critical effect on the treatment of nonunion by promoting osteogenesis and angiogenesis. This promoting effect might be ascribed to the activation of the BMP-2/Smad1/RUNX2 and the HIF-1α/VEGF signalling pathways.

## Introduction

The repair of bone fractures is a regenerative process that comprises inflammation, angiogenesis, stem cell differentiation, osteogenesis and chondrogenesis [1, 2]. Approximately 5–10% of fractures will lead to delayed healing or nonunion [3], both of which require repeated treatments and have a significant influence on the quality of life. Treatment of nonunion is more difficult than fracture. Surgery is the main clinical treatment. Recently, mesenchymal stem cell (MSC) transplantation has been shown to be effective in implementing regenerative abilities in fracture repair [4, 5]. The use of MSCs in the reparative process can be achieved through autocrine and paracrine approaches [4]. However, there are many factors that restrict the application of mesenchymal stem cells, such as immunosuppression and malignant transformation [6, 7].

Exosomes, important players in cell-to-cell communication in normal physiological and pathological conditions, were recently shown to have an important role in tissue repair [8, 9]. These small particles of 30–150 nm are secreted into the extracellular environment by most cells through fusion with the plasma membrane [10, 11]. Exosomes did not induce overt immune reactions when they were administered to xenogeneic animals. Additionally, they do not contain major histocompatibility complex I (MHCI) or MHCII proteins [12, 13]. The contents of exosomes can be protected from destruction by a liposomal membrane. Specific surface ligands on exosomes can bind to target cells, allowing exosomes to deliver RNAs, proteins or cytokines into the target cells to stimulate particular biological functions [14, 15].

Recent studies have shown that the administration of MSC-Exos can promote endogenous angiogenesis [16], myogenesis [17] and osteogenesis [18]. MSC-Exos have been reported to be effective in the bone regeneration of fractures [18, 19]. However, studies of the treatment of nonunion utilizing MSC-Exos have rarely been reported. In our study, exosomes derived from bone marrow mesenchymal stem cells (BMMSC-Exos) were transplanted into the fracture site in a rat model of femoral fracture. The results demonstrated that the bone healing processes were accelerated by enhanced osteogenesis and angiogenesis.

## Materials and methods

### Cell extraction and culture

Three-week-old Wistar rats (Animal Experiment Center of Shandong University, Jinan, China) were sacrificed by injection with Pelltobarbitalum Natricum. The femurs were collected in clean, vertical flow benches. Bone marrow mesenchymal stem cells (BMMSCs) were washed with DMEM/F12 (1:1) medium (HyClone, Logan, Utah, USA) for collecting cells. BMMSCs were cultured in DMEM/F12 (1:1) medium containing 10% foetal bovine serum (FBS) and 1% double antibiotics (penicillin/streptomycin mix) (Gibco, Rockville, MD, USA). The BMMSCs were maintained in an incubator at 37 °C and 5% CO_2_. The culture medium was replaced every 2 days. When the cells were 80% confluent, BMMSCs were digested with trypsin (Gibco, Rockville, MD, USA) for passaging, and BMMSCs in passages 2–5 were used for the experiments.

### Lipid differentiation and osteogenesis of BMMSCs

The BMMSCs were seeded into 6-well plates. Lipid differentiation was induced by DMEM/F12 (1:1) medium containing 10% FBS, 1% double antibiotics, 0.5 mM 3-isobutyl-1-methylxanthine, 0.2 mM indomethacin, 10 μg/mL insulin and 1 μM dexamethasone for 2 days. The next day, cells were cultured with complete medium containing 10 μg/mL insulin. The entire process lasted 14 days. Then, the BMMSCs were stained with oil red O. Osteogenic differentiation was induced by DMEM/F12 (1:1) medium containing 10% foetal bovine serum (FBS), 1% l-glutamine, 10 mM β-phosphoglycerol, 0.25 mM ascorbic acid and 10 nM dexamethasone for 21 days. After that, the cells were stained with alizarin red.

### Identification of BMMSCs surface antigen by flow cytometry

P3 BMMSCs were digested with trypsin and resuspended in DMEM containing 10% FBS. The cells were washed with phosphate-buffered saline (PBS) twice. Subsequently, the cells were incubated with anti-CD11b/c-PE, anti-CD34-PE, anti-CD29-FITC and anti-CD90-FITC antibodies for 30 min. The cell suspension was then centrifuged at 1000 rpm for 5 min. Finally, the cell suspension was transferred into a new detection tube, followed by the detection of cell surface antigen using flow cytometry (BD Biosciences, San Jose, CA, USA).

### Isolation and identification of exosomes

Following a previously described protocol [19], exosomes were isolated from BMMSC supernatant. Then, 80% confluent BMMSCs were cultured for 48 h in a complete medium, and the medium was moved to new tubes for centrifugation at 300*g* for 10 min at 4 °C. The supernatant was then centrifuged at 16500*g* for 30 min at 4 °C to eliminate cellular debris. The cell supernatant was filtered by using a 0.22-μm filter to remove whole cells and excess cellular debris. Afterwards, the supernatant was moved to new tubes for ultracentrifugation at 100000*g* for 70 min at 4 °C to pellet the exosomes. After collecting the precipitate, ultracentrifugation was performed again, and the supernatant without exosomes was collected for follow-up experiments. Exosomes were identified by nanoparticle tracking analysis (NTA), transmission electron microscopy (TEM) and western blotting.

### In vivo animal experiments

Sixty mature male Wistar rats (12 weeks old, 250–300 g) were used for the study. Animals were randomly divided into control, CM-Exo (exosome-depleted conditioned medium) and Exo (exosomes) groups, *n* = 20 in each.

### Surgical procedures

After the rats were anaesthetized with 3% sodium pentobarbital, surgeries were performed. Briefly, a 10-mm lateral skin incision was made, and the right femur was exposed after exposing the muscle by blunt dissection. A transverse osteotomy was then performed using an osteotome and hammer. The periosteum was destroyed by cauterization with a heated needle. A 1.5-mm-diameter needle (length, 45 mm; material, medical stainless steel 317L) was inserted through the medullary cavity of the distal femur. Then, the other tip of the needle was run through the top of the greater trochanter of the femur. During the reduction and fixation of femoral fractures in rats, a 0.4-mm blade was placed by an assistant at the fracture site to maintain the consistency of the length of the nonunion area. Penicillin (100,000 IU/mL, 1 mL/kg) was injected into the abdominal cavity for 3 days.

The fracture site was located and marked through an X-ray device. Equal volumes of PBS (100 μL), complete medium without exosomes (100 μL) and exosomes (100 μL, 10^10^ particles) were injected into the fracture site of the rats in the three groups, respectively (the skin at the fracture site was located and marked when radiographic examinations were performed). The above measures were taken once a week after the nonunion model was successfully completed.

### Radiographic analysis

Radiographs of the right femurs were acquired through an X-ray device on weeks 8, 14 and 20 after the surgery. X-ray images were scored independently by an imaging specialist as previously described [2], Radiographic images were scored as follows: 1, no apparent hard callus; 2, slight intramembranous ossification; 3, hard callus without bridging of the fracture gap and fracture line is apparent; 4, hard callus with bridging of the fracture gap and fracture gap is noticeable; 5, unclear boundary between the newly formed hard callus and existing cortical bone; and 6, remodelling. Microcomputed tomography (micro-CT) imaging was performed by using high-resolution in vivo X-ray microtomograph Skyscan 1176 (Bruker, Madison, WI, USA). Three-dimensional CT images were recreated by NRecon software. The radiographic data analysis of the femurs was measured by computed tomography using CTAn software.

### micro-CT analysis of angiogenesis at the fracture site

The rats were subjected to internal fixation removal 20 weeks after the surgery. The whole vascular system was flushed by injecting heparinized (100 U/mL) normal saline and polyformaldehyde solution. Then, MICROFIL (Flow Tech, Carver, MN, USA) was perfused by intracardiac injection. Next, the animals were maintained at 4 °C for 24 h. The lower right limbs of the animals were scanned using the microtomograph Skyscan 1176.

### Western blot analysis

Total protein from bone tissues around the fracture site was extracted from the control group, CM-Exo group and Exo group 20 weeks after the operation. Bone tissues were derived from the animals and ground into powder in a mortar with liquid nitrogen and then transferred to a pre-cooled centrifugal tube to extract the total protein. Western blotting was performed according to previous studies [20]. The obtained PVDF membrane was incubated overnight with primary antibodies against BMP-2 (1:1000), RUNX2 (1:1000), Smad1 (1:1000), HIF-1α (1:1000), OPN (1:1000), OGN (1:1000) (Cell Signaling Technology, MA, USA) and VEGFA (1:1000) (Abcam, Cambridge, UK) at 4 °C. Finally, the PVDF membranes were incubated in peroxidase-coupled avidin goat anti-rabbit IgG (Cell Signaling Technology, MA, USA) at room temperature for 1 h. Then, the membranes were scanned and protein levels were normalized to β-actin (1:1000) as a control. The ChemiDoc Touch Gel Imaging System and Image Lab Touch software (Bio-Rad, CA, USA) were used to record and quantify the signal intensity.

### PCR

To measure the mRNA expression, total RNA from bone tissues around the fracture site was isolated by using TRIzol (Life Technologies, CA, USA) reagent [21]. The cDNA was reverse transcribed using ReverTra Ace qPCR RT Kit (TOYOBO, Japan). The reaction was performed on a MasterCycler (Eppendorf, GRE). Quantitative real-time PCR (qRT-PCR) was performed in a LightCycler480 System (Roche, CH) using the LightCycler480 software 1.5.1.62 SP3 to determine the mRNA expression levels of various genes. The relative mRNA levels were normalized to those of β-actin mRNA and were evaluated by using the comparative CT method.

### Histological evaluation

The femurs of rats from the control, CM-Exo and Exo groups were harvested 16 weeks after the operation. The samples were immersed in 4% paraformaldehyde at 4 °C for 24 h, decalcified in 10% EDTA at room temperature for 21 days and then embedded in paraffin. The femurs were sectioned (3-μm slices) along the longitudinal axis and stained with toluidine blue, safranin O-fast green and haematoxylin and eosin (H&E) for the analysis of histologic differences. Two different sections of each sample were analysed. Fracture healing was evaluated by using a histological score of fracture healing [22].

### Immunohistochemistry

For IHC analysis, sections from paraffin-embedded femurs were dewaxed by a routine method. The expression of BMP-2, Smad1, RUNX2, OPN, OGN, OCN and CD31 was measured to evaluate osteogenesis and angiogenesis in the femur. The positively stained tissues were visualized under a light microscope and analysed by Image-ProPlus 6.0 software.

#### In vitro studies

##### MC3T3-E1C and HUVEC cell cultures

Mouse embryo osteoblast precursor cells (MC3T3-E1Cs) and human umbilical vein endothelial cells (HUVECs) were purchased from Zhong Qiao Xin Zhou Biotechnology Co., Ltd. (Shanghai, China). MC3T3-E1Cs were cultured in MEM-α medium containing 10% foetal bovine serum (FBS) and 1% double antibiotics. HUVECs were cultured in endothelial cell medium containing 10% FBS, endothelial cell growth supplement and 1% double antibiotics. Both cell types were maintained in an incubator at 37 °C and 5% CO_2_. Cells from the third passage were used in the studies.

##### BMMSC exosome uptake by MC3T3-E1Cs and HUVECs

The isolation of exosomes was similar to that described above. Exosomes were stained with a red fluorescent dye (PKH26, Sigma-Aldrich, USA) as previously described and then incubated with MC3T3-E1Cs and HUVECs at 37 °C for 12 h. The cells were subsequently washed with PBS and fixed in 4% paraformaldehyde for 30 min. After washing with PBS twice, the cells were immersed in 0.5% Triton-X 100 (Solarbio, Beijing, China) and stained with FITC Phalloidin (Solarbio, Beijing, China). The nuclei were stained with DAPI (Solarbio, Beijing, China). A laser scanning confocal microscope LSM780 (Zeiss, GRE) was used to detect the signals in stained cells.

##### Scratch wound assay

Cell migration was evaluated by a scratch wound healing assay. MC3T3-E1Cs and HUVECs were seeded in 6-well plates and grown to confluence. A scratch of approximately 1 mm was created in the confluent cell layer by using a P200 pipette tip. After three washes with PBS to remove loose cells, the control group was treated with medium alone and the test group was treated with 100 μL of exosomes (1 × 10^10^ particles), and the plate was incubated at 37 °C. Images were obtained at 0, 12 and 24 h and measured using ImageJ software.

##### EdU incorporation

To detect the proliferation ability of MC3T3-E1Cs and HUVECs, the cells were seeded in 24-well plates in a suitable medium with 10% FBS for 24 h. Then, the cells were cultured using a serum-free medium, and BMMSC-Exos were added to the test groups. A 1:1000 dilution of EdU-labelling reagent (Ribobio, Guangzhou, China) was added after that. Six hours later, the cells were fixed with paraformaldehyde for 30 min and subsequently immersed in 2 mg/mL glycine solution for 5 min. Then, the cells were incubated with 0.5% TritonX-100 in PBS at room temperature for 20 min. The cells were detected with a Cell-Light EdU Apollo567 In Vitro Kit (Ribobio, Guangzhou, China) following the instruction manual. Images were analysed using ImageJ software.

##### Tube formation assay

After the basement membrane matrix (Matrigel, BD Biosciences, USA) was dissolved at 4 °C, it was infused into 48-well plates for 100 μL in each well. The wells were incubated at 37 °C for 1 h, then 2 × 10^4^ HUVECs were cultured in 200 μL of endothelial cell medium without FBS and added to the plates. The control group was treated with medium alone, and the test group was mixed with 100 μL of exosomes. Tube branches and the total length of the tubes were calculated using ImageJ software.

##### Western blot analysis and immunocytochemistry

To detect protein expression following BMMSC-Exo treatment, MC3T3-E1Cs were seeded into 6-well plates and supplemented with serum-free medium (MEM-α) after 24 h. The cells were separately incubated with BMMSC-Exos, LDN-193189 (10 μM) and noggin (100 ng/mL). After 24 h, cell lysates from one 6-well plate were harvested and maintained on ice for 30 min. The nuclear and cytoplasmic proteins were used for western blotting. The other 6-well plate was used for immunocytochemistry. The methods were performed as before.

##### ALP activity assay and alizarin red S staining

Two groups of MC3T3-E1 cells were plated into 6-well plates and cultured in osteogenic differentiation medium containing different treatments (control group and BMMC-Exo group) for 14 days. The cells were then immobilized, and ALP activity was assayed with an ALP staining kit (Beyotime Institute of Biotechnology, Shanghai, China). Bone mineralization was determined by alizarin red S (Solarbio, Beijing, China) staining at 21 days.

### Statistical analysis

All values are represented as the mean ± standard deviation. SPSS 22.0 software was utilized to perform statistical analyses. All experiments were repeated at least three times. Student’s *t* test was used for comparisons of two independent groups. Analysis of variance was used for the comparisons between multiple groups. *P* values < 0.05 were considered statistically significant.

## Results

### BMMSC phenotype and multidirectional identification

**Fig. 1:**
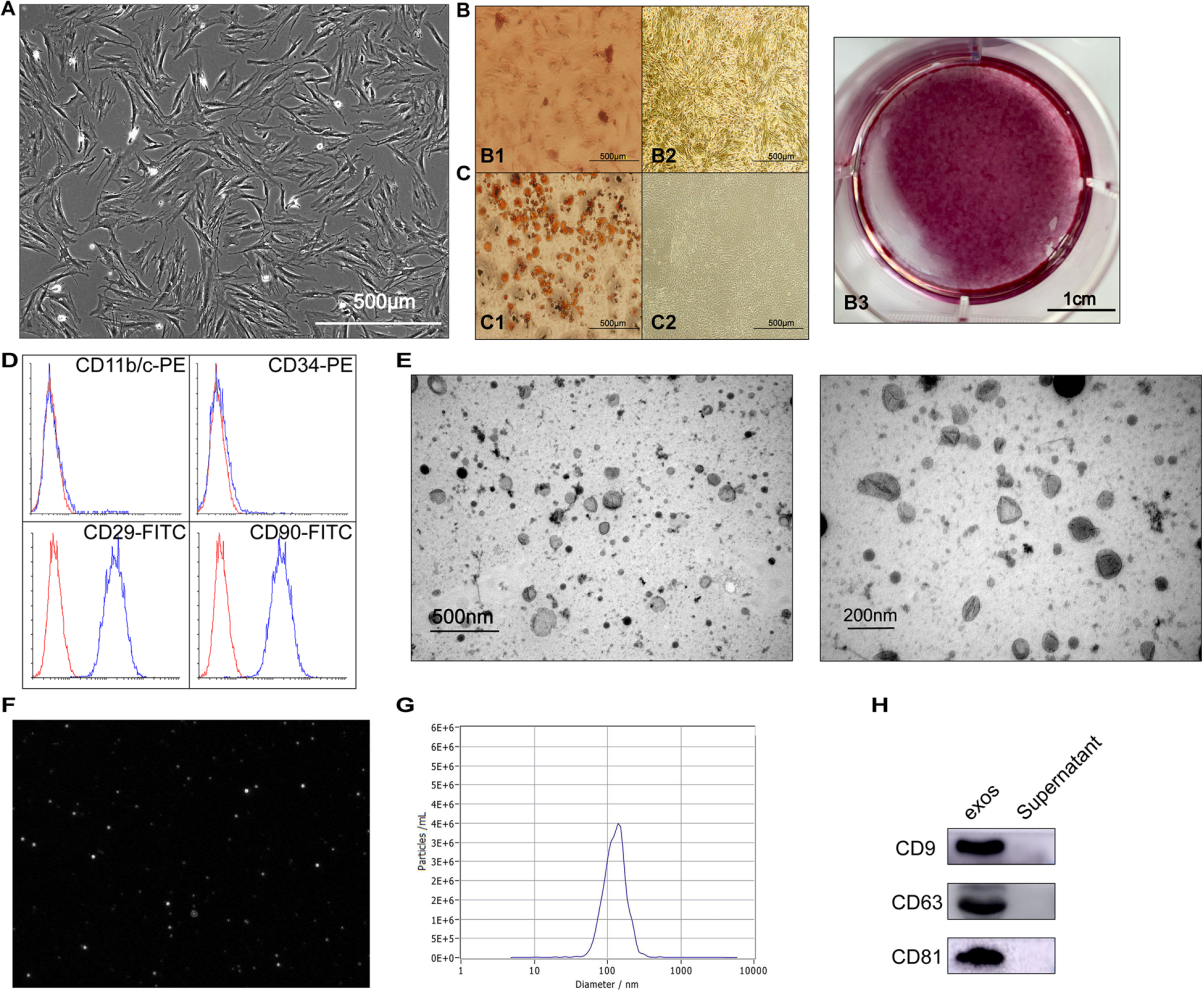
Characterization of BMMSCs and BMMSC-Exos. **a** Fusiform morphology of BMMSCs shown in light microscopy images. **b** Alizarin red staining was performed to detect the osteogenic differentiation ability of BMMSCs: B1, staining of experimental group; B2, staining of control group; B3, gross scanning images of ARS staining of experimental group. **c** Oil red staining was performed to detect the lipid differentiation ability of BMMSCs: C1, staining of the experimental group; C2, staining of the control group. **d** Surface markers of BMMSCs analysed by flow cytometry. The cells were negative for CD34 and CD11b/C and positive for CD90 and CD29. **e** The morphology of BMMSC-Exos shown by TEM. **f** Image of the purified exosomes. **g** The particle size distribution in purified BMMSC-Exos determined by NTA. **h** The surface markers (CD9, CD63 and CD81) of exosomes were detected by western blotting

The BMMSCs extracted from Wistar rats had a fusiform shape and exhibited a vortex distribution (Fig. 1a). Third passage cells were seeded into 6-well plates for induction of osteogenesis and lipid differentiation. After induction for 21 days, alizarin red staining results indicated that there were many calcified nodules (Fig. 1b). Similarly, oil red staining results also showed a very large number of lipid droplets (Fig. 1c). Expression of the cell surface antigens CD11b/C, CD34, CD29 and CD90 was detected by flow cytometry. The results showed that the cells were negative for CD11b/C (< 5%) and CD34 (< 5%) and positive for CD29 (> 95%) and CD90 (> 95%) (Fig. 1d).

### Characterization of exosomes

The extracted exosomes were characterized using TEM, NanoSight and western blotting. TEM images showed that the majority of the particles exhibited a cup- or round-shaped morphology. The diameter of the exosomes was approximately 122 nm (Fig. 1g). The expression of the CD9, CD63 and CD81 proteins was detected (Fig. 1h). The results indicated that the extracted exosomes had characteristics in accordance with widely accepted standards.

### X-ray analysis of fracture healing

**Fig. 2:**
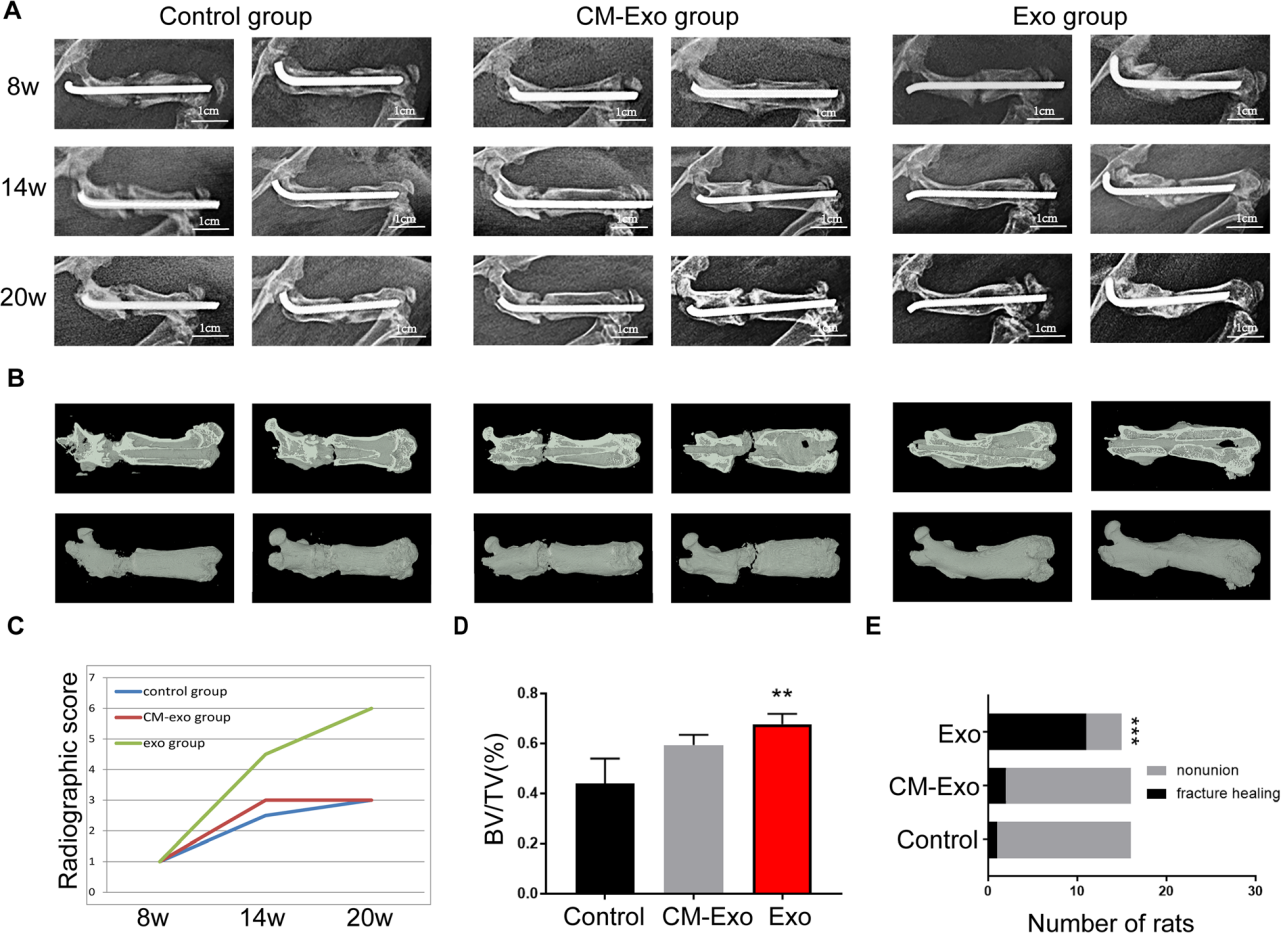
Radiographic analysis of fracture healing. **a** Representative radiological images of the control, CM-Exo and Exo groups at 8, 14 and 20 weeks after surgery. (Radiological examination 8 weeks after the surgery was performed to confirm successful nonunion modelling). **b** Representative micro-CT images of a nonunion femur at 20 weeks after surgery. **c** Radiographic score for X-ray images on postoperative weeks 8, 14 and 20, *n* = 5. **d** BV/TV on postoperative week 20 was quantified, *n* = 3. **e** Bone union rate was calculated by chi-square test in the control, CM-Exo and Exo groups. Abbreviations: 8w, 8 weeks; CM, conditioned medium; CM-Exo, exosome-depleted conditioned medium; Exo, exosomes; BV, bone volume; TV, total volume. **P < 0.01; ***P < 0.001

X-ray images of rats in all three groups were taken to confirm whether the nonunion models were successfully established 8 weeks after the surgery. In addition to the severe infection of 2 rats, comminuted fractures of 3 rats, internal fixation failure of 7 rats and 1 death, the other 47 rats succeeded in establishing the nonunion model, and 16 rats were included in the control group, 16 in the CM-Exo group, and 15 in the Exo group. It can be observed in the X-ray images that primary callus and hard callus appeared in the rats of the Exo group on postoperative weeks 14 and 20 (Fig. 2a). In addition, the fracture gap was obvious in the other two groups, and almost no callus was observed in most rats in the control and CM-Exo groups. Quantitative analysis of the radiographic score showed significantly higher values in the Exo group than in the other two groups on postoperative week 20 (Fig. 2c).

### micro-CT analysis of fracture healing

For micro-CT analysis, a region of interest (ROI) in the fracture site was selected for statistical analysis and observation. In the control and CM-Exo groups, the fracture gap became clearer, and the bone in these areas had vanished. In the Exo group, the images revealed the morphology of a newly formed bone in the fracture gap (Fig. 2b). Quantitative analysis showed that BV/TV was significantly higher in the Exo group than in the control and CM-Exo groups (*P* < 0.01 for all variables, Fig. 2d).

### Histological analysis

**Fig. 3:**
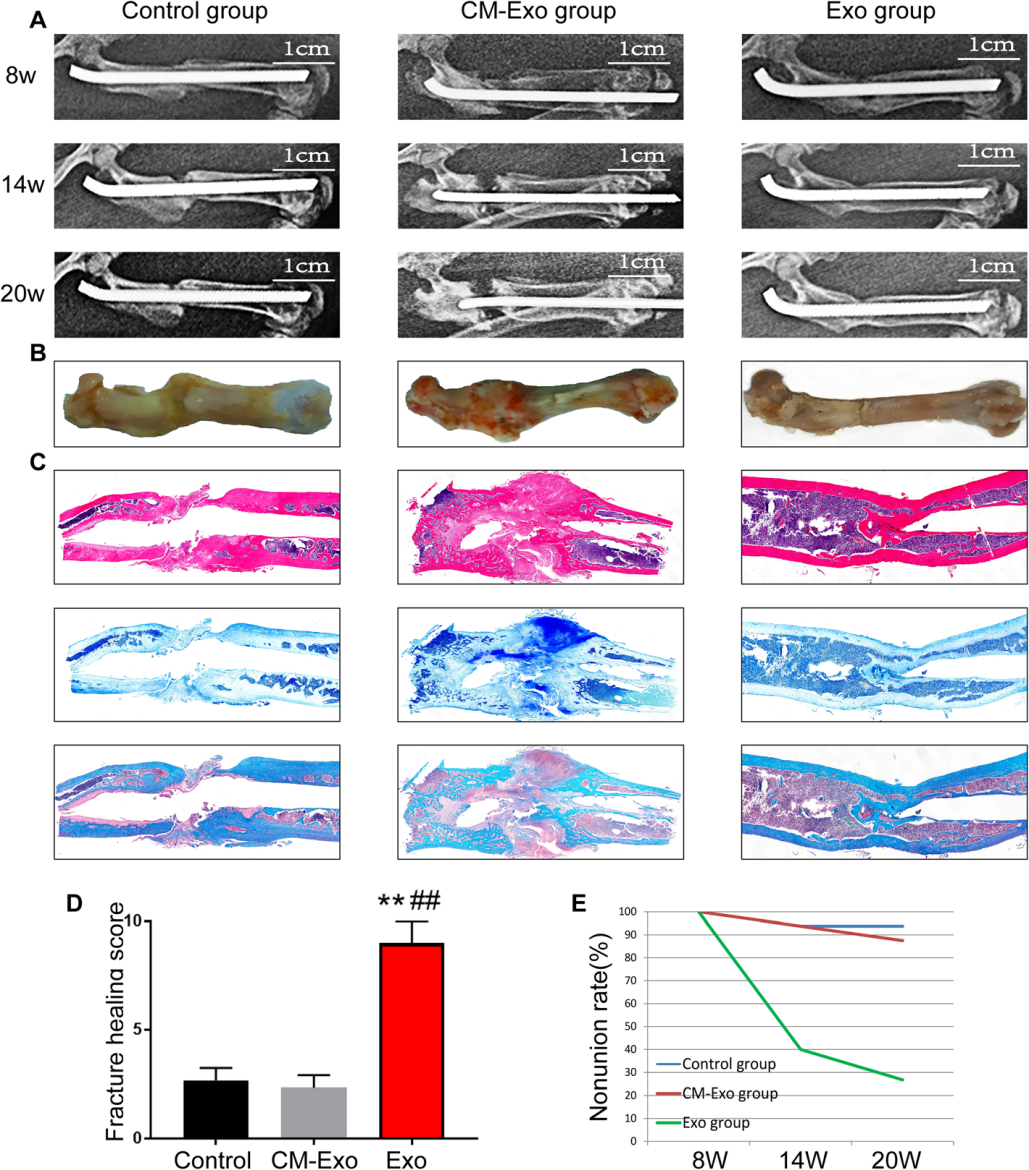
Radiographic and histological analysis of femoral specimens. **a**, **b** Radiographic imaging by X-ray was performed at 8, 14 and 20 weeks after the surgery, and rat femurs were harvested at 20 weeks after the surgery. **c** H&E, toluidine blue and safranin O-fast green staining of the femurs. **d** Quantification of fracture healing in calluses. **e** Bone nonunion rate of the control, CM-Exo and Exo groups. Abbreviations: 8w, 8 weeks; H&E, haematoxylin-eosin. **Statistically significant difference compared with the control group (*P* < 0.01). ^##^Statistically significant difference compared with the CM-Exo group (*P* < 0.01)

Histological analysis with H&E, toluidine blue and safranin O-fast green staining revealed that there was a visible improvement in the fracture healing of the femur in exosome-injected rats compared with rats in the other two groups (Fig. 3c). In addition, a woven bone was observed in the fracture site of the femur in the Exo group. Moreover, there was substantial fibrous tissue and synostosis in the fracture site of the femur in the control and CM-Exo groups.

### BMMSC-Exos enhance angiogenesis in the bone tissue at the fracture site

**Fig. 4:**
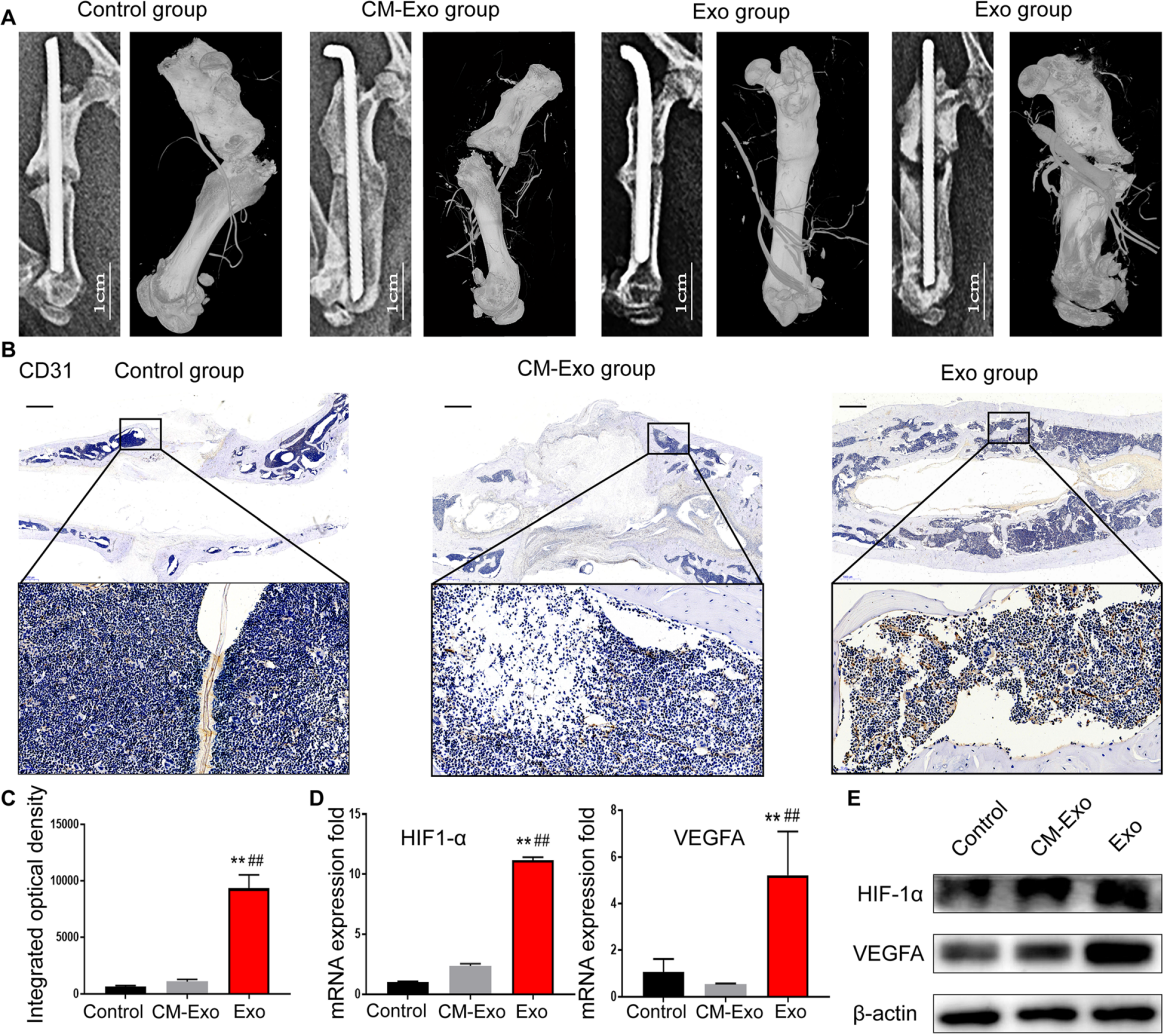
Assessment of angiogenesis at the fracture site. **a** Representative micro-CT images of the vasculature in the fracture site on postoperative week 20. **b** Anti-CD31 staining of the femur slices on postoperative week 20. Representative areas are shown, and boxed areas are enlarged on the bottom. Scale bar = 1 mm. **c** The integrated optical density of CD31-positive vessels was measured. **d** Total RNA was extracted, and the expression levels of osteogenesis-related genes (HIF1-α and VEGF) in the bone tissue of the fracture sites were analysed by qRT-PCR, *n* = 3. **e** Western blot analysis showed that HIF-1α and VEGF protein levels increased in the bone tissue of the fracture sites stimulated with BMMSC-Exos. Abbreviations: CM, conditioned medium; CM-Exo, exosome-depleted conditioned medium; Exo, exosomes. **P < 0.01 vs the control group; ^##^*P* < 0.01 vs the CM-Exo group

Angiogenesis of the bone at the fracture site was evaluated by 3D microangiography and IHC staining. The micro-CT images revealed significantly more vascular branches in the fracture site in the Exo group compared with the control and CM-Exo groups (Fig. 4a). The comparison of vessel volume was also consistent with the above observation.

Platelet endothelial cell adhesion molecule-1 (CD31) participates in the process of angiogenesis. The results of IHC staining showed increased expression of CD31 in the target bone tissue of the Exo group compared with those in the other two groups (Fig. 4b). Integral optical density (IOD) was calculated following IHC staining by Image-ProPlus 6.0 software. In addition, higher IOD scores in the target area were obtained for the Exo group (Fig. 4c), and there were significantly more CD31-positive blood vessels after the administration of BMSC-Exos.

Evidence indicates that the growth of blood vessels in the bone is critical to fracture healing [23]. As an important proangiogenic cytokine, vascular endothelial growth factor (VEGF) plays a key role in the process of vascular development and regeneration. The mRNA and protein expression levels of VEGF and HIF-1α in the bone tissue of the fracture site were detected by qRT-PCR and western blotting. The results showed that the levels of VEGF and HIF-1α were increased in the Exo group compared with the control and CM-Exo groups (Fig. 4d). These results demonstrated that HIF-1α-VEGF signalling was activated by BMMSC-Exos.

### BMMSC-Exos enhance osteogenesis in vivo

**Fig. 5:**
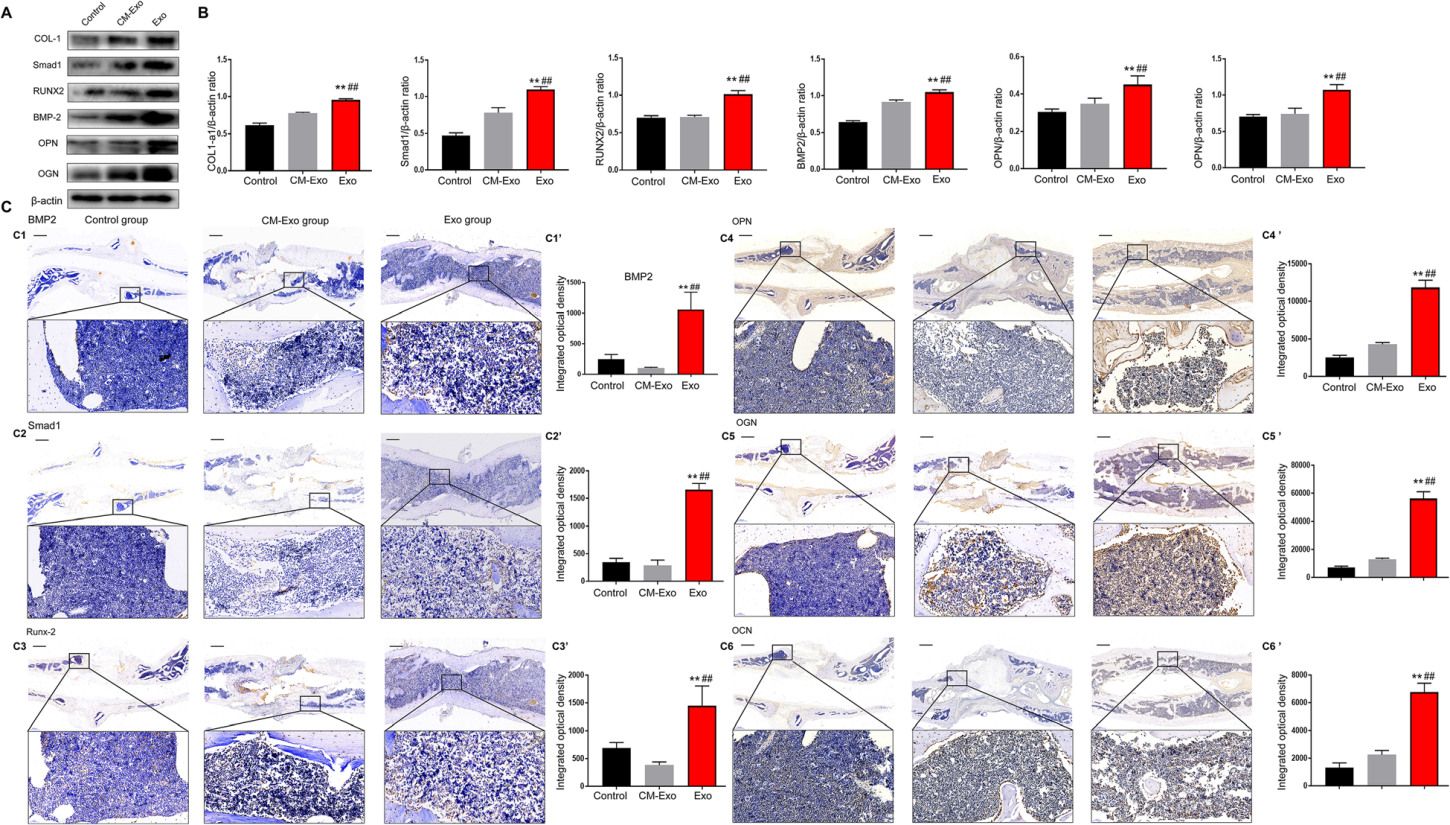
Assessment of osteogenesis in the bone tissue. **a** Western blot analysis showed that the Smad1, RUNX2, BMP-2, OPN and OGN protein levels increased in the bone tissue of the fracture sites stimulated with BMMSC-Exos. **b** The grey value ratios were quantified using ImageJ software (NIH, USA). The experiments were performed on three separate occasions. **c** The immunohistochemical analysis of BMMSC-Exos promoted osteogenesis in vivo. Immunohistochemical analysis of BMP-2 (C1), Smad1 (C2), RUNX2 (C3), OPN (C4), OGN (C5) and OCN (C6) was used to detect osteogenesis in the femur specimens. The brown colour represents positive staining of BMP-2, Smad1, RUNX2, OPN, OGN and OCN. The integrated optical density of positive staining was measured (C1′–C6′). Abbreviations: CM, conditioned medium; CM-Exo, exosome-depleted conditioned medium; Exo, exosomes. **P < 0.01 vs the control group; ^##^*P* < 0.01 vs the CM-Exo group

Previous studies have indicated that the BMP-2/Smad1/Runx2 pathway is involved in the proliferation and osteogenic differentiation of BMMSCs [24]. Proteins of the BMP-2/Smad1/RUNX2 signalling pathway were evaluated by western blotting. The results revealed that the levels of BMP-2, Smad1 and RUNX2 were increased in the Exo group. This finding supported the idea that BMMSC-Exos significantly influenced BMP-2/Smad1/RUNX2 signalling in vivo. Moreover, the expression of OPN and OGN was also increased (Fig. 5a). The western blotting results identified an increased expression level of osteogenesis-related genes.

The IHC results demonstrated an increased expression of BMP2, Smad1/5, RUNX2, OGN, OPN and OCN in the target bone tissue of rats in the Exo group compared with the control and CM-Exo groups (Fig. 5c), and higher IOD scores in the fracture site were obtained for the Exo group.

### BMMSC-Exos promote the proliferation and migration of HUVECs and MC3T3-E1Cs in vitro

**Fig. 6:**
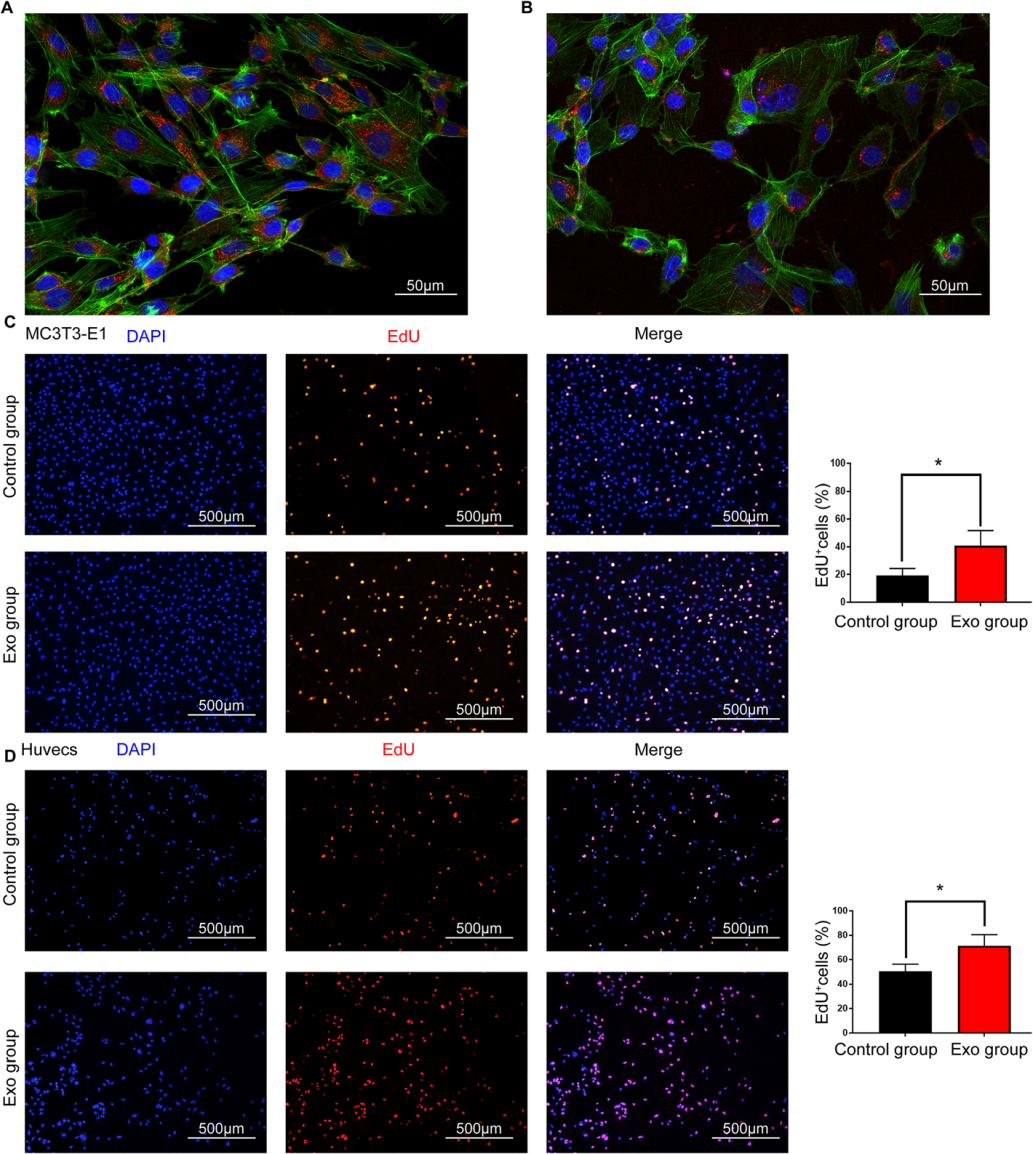
BMMSC-Exos entered MC3T3-E1Cs and HUVECs and promoted the proliferation of the cells. **a**, **b** Laser scanning confocal microscopy analysis of the internalization of PKH26-labelled BMMSC-Exos by MC3T3-E1Cs (**a**) and HUVECs (**b**). The red-labelled exosomes were visible in the perinuclear region of recipient cells. **c**, **d** EdU incorporation by the control and Exo groups of MC3T3-E1Cs (**c**) and HUVCs (**d**) was visualized using a fluorescence microscope. The percentage of EdU-positive cells for each group was quantitated using ImageJ software (right graph), *n* = 3. Abbreviation: Exo, exosomes. *P < 0.05 vs the control group

The ability of exosomes to fuse with cells was verified using the PKH26 assay. BMMSC-Exos were added to HUVECs and MC3T3-E1Cs after staining. Laser scanning confocal microscopy analysis indicated that cells treated with stained exosomes presented PKH26 cytoplasmic fluorescence (Fig. 6a). This finding revealed that the purified exosomes were able to be internalized.

**Fig. 7:**
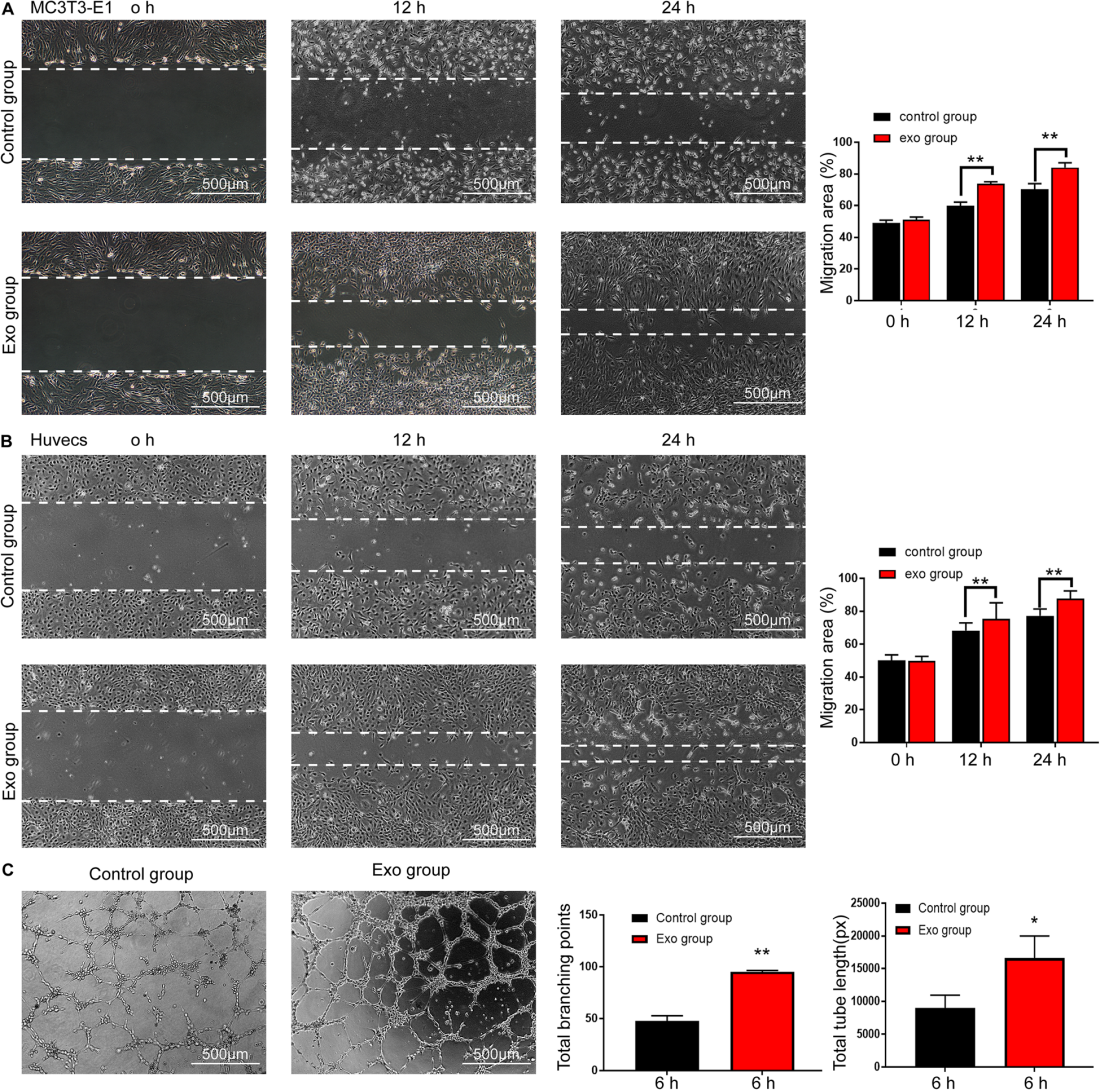
BMMSC-Exos enhanced the migration ability and angiogenesis of MC3T3-E1Cs and HUVECs. **a**, **b** Scratched wound assay and quantitative analysis of MC3T3-E1Cs and HUVECs. The migration area was significantly greater in the Exo group than in the control group at 12 h and 24 h. **c** BMMSC-Exos stimulated the tube formation ability of HUVECs, and quantitative analysis is shown in the graphs on the right. Abbreviation: Exo, exosomes. *P < 0.05 vs the control group; **P < 0.01 vs the Control group

EdU test and scratch wound assay were performed to detect cell proliferation and migration capabilities. The EdU test demonstrated that, compared with those in the control group, there was a significantly higher percentage of EdU-positive cells in the Exo group (Fig. 6b, c). The migration capacity of HUVECs and MC3T3-E1Cs was enhanced at 12 and 24 h after exosomes were added, and the migration area was markedly increased in the Exo group compared with the control group (Fig. 7a).

### Tube formations of HUVECs are promoted by exosomes in vitro

A tube formation assay in HUVECs showed that BMMSC-Exos significantly enhanced the angiogenic tube formation ability of HUVECs (Fig. 7c). The values of tube lengths and branch points were obviously higher in the Exo group compared with the control group.

### BMMSC-Exos promote the osteogenic ability of MC3T3-E1Cs in vitro

**Fig. 8:**
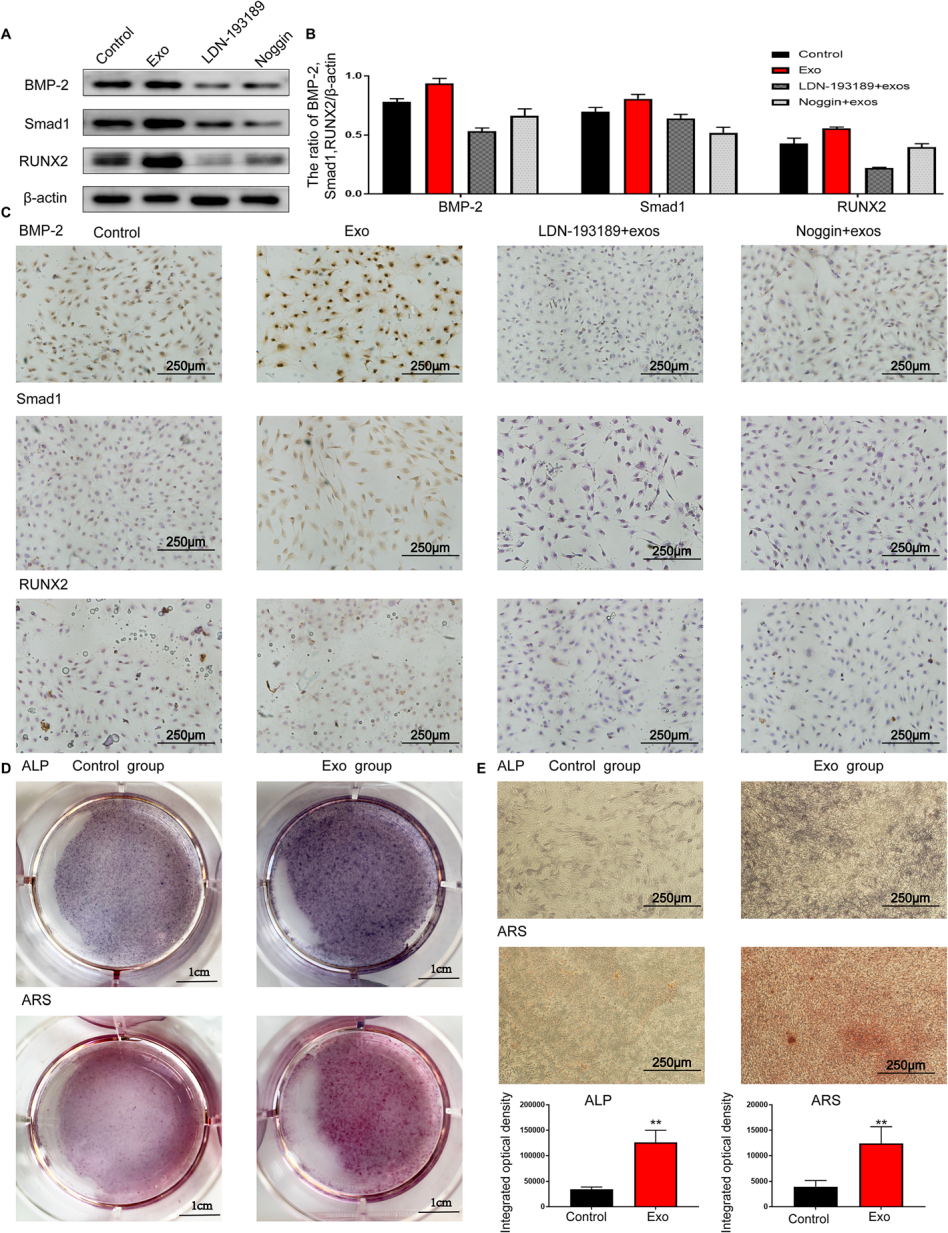
The inhibition of BMP-2 decreased the Smad1 and RUNX2 protein levels in MC3T3-E1Cs stimulated with BMMSC-Exos. **a** Western blot analysis showed that even if BMMSC-Exos were applied, the expression levels of BMP-2, Smad1 and RUNX2 proteins were markedly increased after BMP-2 was inhibited. **b** The grey value ratios were quantified. **c** The immunocytochemistry analysis showed that applying BMMSC-Exos+BMP-2 inhibitor could not promote osteogenesis. **d** Gross scanning images of ALP and ARS staining of MC3T3-E1Cs (control group and BMMSC-Exos group), ALP staining at 14 days and ARS staining at 21 days. **e** Quantitative analysis of ALP and ARS activity was used to evaluate the effect of BMMSC-Exos on the osteogenic ability of MC3T3-E1Cs

The BMMSC-Exos were added to MC3T3-E1Cs 6-well plates to quantify ALP activity as a marker of osteogenic ability. As shown in Fig. 8d, e, more intensive ALP staining was observed in cells treated with BMMSC-Exos compared with the control group. Quantitative analysis of ALP activity showed that there was a significant difference between the two groups (*P* < 0.01) at 14 days. The results of alizarin red S staining showed that the deposition of the mineral was significantly enhanced compared with the control group at 21 days (Fig. 8d, e). The results showed that BMMSC-Exos promoted the osteogenic ability of MC3T3-E1Cs.

### BMMSC-Exos failed to enhance osteogenesis in vitro after the action of BMP2 inhibitors

After treatment with BMMSC-Exos for 24 h, the expression levels of Smad1 and RUNX2 protein in MC3T3-E1Cs were markedly increased. When cells were incubated with BMMSC-Exos in the presence of the BMP2 inhibitors LDN193189 or noggin, the stimulatory effect of the exosomes on the expression of Smad1 and RUNX2 protein was significantly reduced both in western blotting and immunocytochemistry assays (Fig. 8). The results indicated that BMMSC-Exos activated osteogenic differentiation through the BMP-2/Smad1/RUNX2 signalling pathway.

## Discussion

Nonunion is a problem that may occur following bone fractures or large segmental bone defects and results in a decreased quality of life for patients. Surgery is the main method of clinical treatment, and surgical options include refixation, solitary bone grafting and osteotomy (with or without bone grafting) [25–28]. In recent years, stem cell transplantation has been an alternative option for the treatment of bone nonunion [3, 29, 30]. MSCs are considered one of the most suitable types of stem cells for autologous transplantation therapy because of their bone regeneration potential [31, 32]. BMMSC transplantation has been proven effective in enhancing osteogenesis and angiogenesis [33]. Baker et al. revealed that human MSC osteogenesis occurs through the PI3K/Akt signalling pathway [34]. Ismail et al. indicated that the combination of autologous BMMSCs and HA granules was a safe method for the treatment of nonunion [35]. Nevertheless, the application of stem cells is still restricted by immunological rejection, malignant transformation, chromosomal variation and so on [36]. In addition, most MSCs used for clinical trials lack sufficient preclinical studies and manufacturing quality control [34].

As principal mediators of intercellular communication with cells, exosomes are important paracrine factors that can be used as therapeutic means for tissue repair, especially in the field of bone regeneration [19, 37]. Compared with stem cells, exosomes do not induce overt immune reactions, even if they are administered to nonimmune-compatible animals [38, 39]. Zhao et al. showed that BMMSC-Exos could improve osteoporosis in vitro [40]. Narayanan et al. indicated that exosomes could induce specific differentiation of naive MSCs in vitro and in vivo and could promote vascularization in the fracture healing process [41]. Exosomes are well tolerated in the body as evidenced by their wide distribution in biological fluids [42]. Our study suggests for the first time that BMMSC-Exos could promote angiogenesis and osteogenesis in a rat nonunion model. The in vitro results revealed that BMMSC-Exos might increase osteogenic differentiation through the activation of the BMP-2/Smad1/RUNX2 signalling pathway. In addition, the functions of vascular endothelial cells were also promoted.

In nonunions, the pool of BMMSCs is decreased, and their proliferation is delayed [43]. However, BMMSCs from nonunions are able to proliferate, differentiate into osteoblastic cells and mineralize in vitro under appropriate conditions [44]. Exosomes can transfer proteins, mRNAs and microRNAs as carriers between different cells, and the gene expression and protein translation of the recipient cells can be altered by the delivered cargo. The bioactivity of target cells can be regulated by such intracellular transport [45]. Exosomes can function in the target area due to their characteristics, including physical properties such as stability, biocompatibility, permeability, low toxicity and low immunogenicity [46]. Our study provides evidence that BMMSC-Exos could strongly enhance bone regeneration. In vitro and vivo, the expression levels of osteogenesis-related genes were analysed by IHC and western blotting. The results showed that OPN and OGN expression levels were significantly increased. IHC analysis verified the increased expression of OCN, OPN and OGN in target bone tissue stimulated by BMMSC-Exos.

The BMP-2/Smad1/RUNX2 signalling pathway is associated with osteogenesis, and RUNX2 is a key factor in bone formation [47]. Smad proteins interact with RUNX2 to participate in the expression and differentiation of osteoblast phenotype genes. RUNX2 and Smad proteins co-regulate the expression of collagen in osteoblasts [48]. Our study demonstrated that the expression levels of BMP-2, Smad1 and RUNX2 were markedly increased in callus tissue and MC3T3-E1Cs stimulated by BMMSC-Exos using western blotting, IHC and ICC. However, the expression levels of these genes were decreased in vitro when the BMP2 inhibitors noggin and LDN193189 were added. In addition, this result illustrates that this signalling pathway plays an important role in the effectiveness of BMMSC-Exos to enhance osteogenesis.

Fracture healing relies on the formation of new blood vessels in the fracture site, and angiogenic factors have been considered indispensable in this process [49, 50]. Hypoxia-inducible factor-1α (HIF-1α) was demonstrated to be a pivotal regulator of angiogenic-osteogenic coupling during bone regeneration [51]. Moreover, the proliferation, migration and tube formation of endothelial cells are instrumental to angiogenesis in fracture healing [51]. The in vivo results suggested that BMMSC-Exos could visibly enhance angiogenesis. The expression levels of angiogenesis-related genes in target tissues stimulated by BMMSC-Exos were analysed by RT-PCR and western blotting, and HIF-1α and VEGF were demonstrated to be significantly increased. The in vitro results showed that the abilities of HUVECs stimulated by BMMSC-Exos to proliferate, migrate and form tubes were significantly enhanced.

However, there are several limitations to the current study. The clinical treatment of exosomes for nonunion has not been developed; surgery cannot be replaced by this method temporarily. In clinical nonunion, usually surgical debridement and readjustment of fixation are needed, which is different from the animal nonunion model.

## Conclusions

**Fig. 9:**
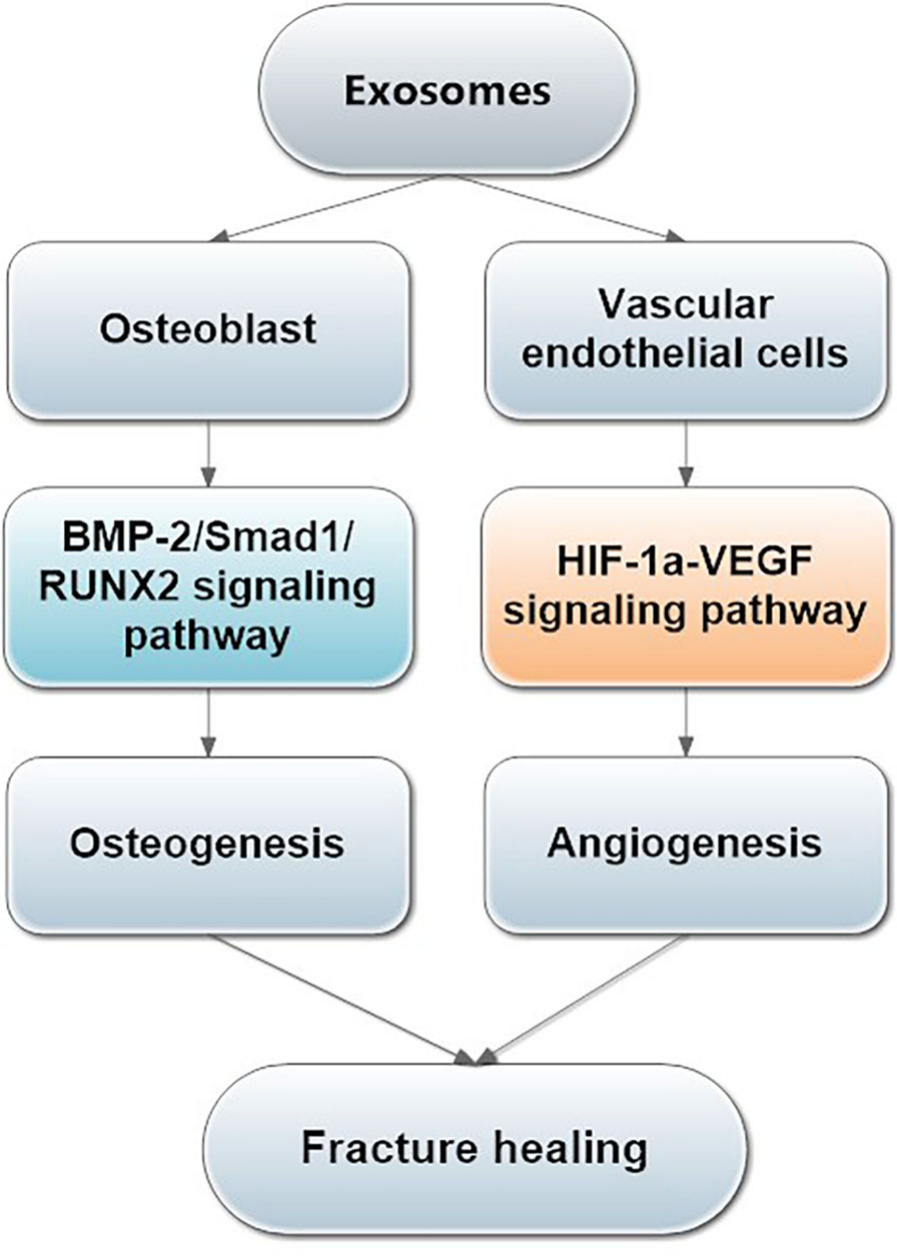
Research schematic. BMMSC-Exos could accelerate the proliferation and migration of osteoblast cells and endothelial cells, further promoting angiogenesis and osteogenesis by activating the HIF-1α/VEGF and the BMP-2/Smad1/RUNX2 signalling pathways to enhance fracture healing

In summary, our current study demonstrated that BMMSC-Exos could accelerate the proliferation and migration of endothelial cells and osteoblast cells, further promoting angiogenesis and osteogenesis to enhance fracture healing(Fig. 9). As an intercellular communicator, exosomes can activate the HIF-1α/VEGF and the BMP-2/Smad1/RUNX2 signalling pathways, and this may be one of the underlying mechanisms in the fracture healing process. The data presented here provide the first evidence for the potential of BMMSC-Exos in treating nonunion. Our findings suggest that BMMSC-Exos may be a prospective therapeutic approach in the treatment of nonunion.

## Data Availability

The datasets generated during the current study are available in the figshare repository. http://doi.org/10.6084/m9.figshare.8965286.

## Abbreviations

MSC: Mesenchymal stem cell
exo: Exosomes
BMMSCs: Bone marrow mesenchymal stem cells
BMMSC-Exos: Exosomes derived from bone marrow mesenchymal stem cells
CM-Exo: Exosome-depleted conditioned medium
FBS: Foetal bovine serum
MHC: Major histocompatibility complex
NTA: Nanoparticle tracking analysis
TEM: Transmission electron microscopy
qRT-PCR: Quantitative real-time PCR
MC3T3-E1Cs: Mouse embryo osteoblast precursor cells
HUVECs: Human umbilical vein endothelial cells
